# Chloretone as an Hypnotic and Local Anæsthetic

**Published:** 1901-06

**Authors:** 


### Reviews of Dental Literature.

---

Chloretone as an Hypnotic and Local Anæsthetic.—*Medicine* for August, 1900, contains an article by Ward upon this important and useful new drug. As he well says at the beginning of his article, hypnotics at best are only palliative, and are useful chiefly to tide patients over a critical period until they can be put into a condition to obtain a natural and normal amount of sleep. They perform a useful office by aiding us to put patients in a physical condition in which they will need no such artificial aid. An ideal hypnotic would be one which would produce sleep without disturbance of the digestion, circulation, or other unpleasant sequelæ. We have hypnotics which come very close to meeting these conditions, but there are none of them which are harmless if they are used for a long time and in quite large doses. One can but condemn the freedom with which the laity purchase the so-called harmless hypnotics. Ward has known of at least two deaths from twenty-grain doses of phenacetine taken upon the patient's own responsibility. Drugs of this character can be purchased as freely as coffee, and he has no doubt that if the experience of the profession could be recorded it would furnish an appalling array of disasters due to the enormous consumption of sulphonal and trional by the laity, without professional advice.

Ward has personally experimented upon himself with a number of the later hypnotics to observe their effects. Among these, chloretone was found to be the most efficient and the freest from bad after-effects. Next to chloretone, trional is one of the best hypnotics. Sulphonal may often be administered with excellent effect, but the bad results which sometimes follow its administration make one fearful in its use. Upon himself, trional caused a slight dizziness, which was noticeable the following day. In patients this was noticed only occasionally. After taking chloretone for several nights in succession he noticed in the early morning a slight headache, which disappeared after taking a cup of coffee. He is not sure that this headache should be attributed to the drug, but not having been subject to it, it may be fairly charged to the chloretone.

After employing chloretone upon himself, Ward gave it freely to quite a large number of cases in which sleep was indicated. None of the patients knew what they were taking. One of the most brilliant results was in the case of a woman eighty-four years old, who, as a result of domestic bereavement, was unable to sleep more than one or two hours at night. This is but one of many such cases, and in only two patients has there been complaint of the slight headache on awakening in the morning.

Chloretone is a product of the decomposition of equal parts of chloroform and acetone in the presence of caustic potash. For a more exhaustive description of the chemical composition and physiological action, the reader is referred to the article by W. H. Porter in the *Post-Graduate* for May, 1900. Water dissolves one per cent. at body temperature. It is not very soluble in gastric juice, but five grains are immediately dissolved by one drachm of alcohol. The best method of administration is to give from three to five grains dissolved in alcohol or whiskey, and follow with a glass of milk at bedtime.

Hypnotics generally have been attracting unusual attention of late, in connection with the treatment of various diseases. The neuron theory of the production of sleep, as developed by Dercum and others, is of especial interest in this connection. It is too much to say that this theory has been proved, but it furnishes an admirable explanation of certain phenomena, and appeals strongly to scientific common sense. It may be said to stand in the same position at the present time to neurology that La Place's nebular hypothesis stood in its earliest days to astronomy, and that Franklin's theories in the kite-flying experiments stood to electricity. The primary features of the neuron theory regard consciousness as having its seat in the cortex of the brain. A theory of sleep based upon this conception regards both the nerve-cells and the dendrites as movable to a limited degree. Consciousness being the sum total of concepts due to the activity of these structures, it follows that as dendrites retract there is a lessening of such concepts with a consequent narrowing of the field of consciousness, until it passes into normal sleep. If this theory of sleep is correct, it follows that drugs such as we are considering have their action explained by their effect upon the dendrites of the cortical cells. The superior dendrites are said by Leach to be depressed by the alcohol radicals of the ethyl and methyl type, while the inferior filaments most readily respond to the depressing effects of chlorine. The hypnotic agent which theoretically should have the best effect is one that would combine chlorine and methyl alcohol and at the same time have in its composition a minimum of other agents which may exert an unfavorable effect. In chloretone we have an hypnotic agent which seems to present these ideal theoretical advantages. It has been tested for some years at the Johns Hopkins Hospital, and bids fair to come into general use as an ideal hypnotic.

Chloretone has served a useful purpose in a number of cases of excessive hyperacidity of nervous origin. There are a class of cases which seriously tax the resources of the physician, and in a number of such cases Ward has given chloral hydrate and belladonna with marked relief. This led to his employing chloretone, as it is a local anæsthetic of some value. It was given in the form of a powder in three- to five-grain capsules on going to bed. It was found to be especially useful in those intractable cases of pain in the epigastrium which come on at night, interrupt sleep, and sometimes cause the patient to walk the floor, and to take large doses of bicarbonate of soda. The drug was used empirically, and its beneficial effect is undoubtedly to be attributed to its local anæsthetic action upon the gastric mucous membrane.

Chloretone would seem to be an ideal remedy in acute alcoholism, as we would not only obtain its hypnotic effect, but also its sedative action upon an irritable gastric mucous membrane. In such cases chloretone should be given in ten-grain doses dissolved in a half-ounce of whiskey or brandy, this to be followed in a quarter of an hour with a raw egg beaten in a glass of milk. This should be repeated each two hours until the patient is asleep.

Ward has used chloretone in one case of whooping-cough with marked benefit. A child four years of age was given five grains of chloretone dissolved in a teaspoonful of brandy; one-half of this dose was given at the beginning of the attack of coughing at night, and repeated if necessary. The first dose relieved the paroxysm and was followed by a quiet night's rest.—*The Therapeutic Gazette.*

